# Functional Organization of *hsp70* Cluster in Camel (*Camelus dromedarius*) and Other Mammals

**DOI:** 10.1371/journal.pone.0027205

**Published:** 2011-11-09

**Authors:** David G. Garbuz, Lubov N. Astakhova, Olga G. Zatsepina, Irina R. Arkhipova, Eugene Nudler, Michael B. Evgen'ev

**Affiliations:** 1 Engelhardt Institute of Molecular Biology, RAS, Moscow, Russia; 2 Marine Biological Laboratory, Woods Hole, Massachusetts, United States of America; 3 Department of Biochemistry, New York University School of Medicine, New York, New York United States of America; 4 Institute of Cell Biophysics, RAS, Pushchino, Russia; Virginia Tech Virginia, United States of America

## Abstract

Heat shock protein 70 (Hsp70) is a molecular chaperone providing tolerance to heat and other challenges at the cellular and organismal levels. We sequenced a genomic cluster containing three *hsp70* family genes linked with major histocompatibility complex (MHC) class III region from an extremely heat tolerant animal, camel (*Camelus dromedarius*). Two *hsp70* family genes comprising the cluster contain heat shock elements (HSEs), while the third gene lacks HSEs and should not be induced by heat shock. Comparison of the camel *hsp70* cluster with the corresponding regions from several mammalian species revealed similar organization of genes forming the cluster. Specifically, the two heat inducible *hsp70* genes are arranged in tandem, while the third constitutively expressed *hsp70* family member is present in inverted orientation. Comparison of regulatory regions of *hsp70* genes from camel and other mammals demonstrates that transcription factor matches with highest significance are located in the highly conserved 250-bp upstream region and correspond to HSEs followed by NF-Y and Sp1 binding sites. The high degree of sequence conservation leaves little room for putative camel-specific regulatory elements. Surprisingly, RT-PCR and 5′/3′-RACE analysis demonstrated that all three *hsp70* genes are expressed in camel's muscle and blood cells not only after heat shock, but under normal physiological conditions as well, and may account for tolerance of camel cells to extreme environmental conditions. A high degree of evolutionary conservation observed for the *hsp70* cluster always linked with MHC locus in mammals suggests an important role of such organization for coordinated functioning of these vital genes.

## Introduction

Among multiple changes in cellular activity and physiology, the most remarkable event in stressed cells of all organisms studied so far is the rapid production of a highly conserved set of stress proteins usually termed “Heat Shock Proteins” or Hsps because these proteins were originally described in *Drosophila melanogaster* after temperature elevation [1]. Hsps are broadly classified based on their molecular weights and specific functions, and there are several excellent reviews on Hsps classification and function [2]–[4]. In our previous work we concentrated mainly on the role of Hsp70 family in cellular and whole body adaptation of diverse animals to high temperature and other extreme environmental factors [5], [6]. There is a wealth of experimental data suggesting that members of Hsp70 family play an important role in whole body adaptation of animals to adverse environmental conditions [3], [7], [8]. It should be emphasized that the Hsp70 family is most diverse and includes many stress-inducible as well as constitutive proteins playing various roles in different cell compartments and under different cellular conditions [4], [8], [9].

After the discovery of heat shock proteins in Drosophila, we decided to investigate whether there is a correlation between the general pattern of Hsps synthesis and the whole body adaptation in various animals inhabiting thermally contrasting conditions. In our studies we usually compared Hsps synthesis in close species existing under conditions that differ sharply in mean temperature and other parameters of their ecological niches [5]–[7].

Specifically, it has been demonstrated that in poikilothermal organisms high constitutive thermotolerance usually correlates with high contents of Hsp70 in the cells under normal physiological conditions, while inducible thermotolerance develops due to the accumulation of Hsps and especially Hsp70 after temperature elevation [3], [7], [10].

Our studies of heat shock response (HS) were not restricted to insects and other poikilothermic organisms. Previously, we investigated protein synthesis in different human tribes and in camel *Camelus dromedarius* [6], [11]. Camel is a homoiothermal organism perfectly adapted to extreme conditions of arid zone, while its tolerance to heat is accompanied by a significant elevation of the whole body temperature [12]. Previously, one of us (ME) investigated by 2D electrophoresis Hsp70 family proteins in the camel and demonstrated constitutive and differential synthesis of these proteins in cells of different origin [6]. Furthermore, comparison of S^35^-methionine incorporation into the proteins of camel and human lymphocytes at different temperatures showed that camel cells incorporate significantly more label at extreme temperature [6]. Subsequently, other authors demonstrated that unlike fibroblast cells isolated from mice (L929), camel fibroblasts are more resistant to high temperature. Camel cells survive 42°C heat stress in a time-dependent manner and even show growth on par with those cells that were kept at the control temperature of 37°C [13].

Keeping all these data in mind, we continued our studies in order to reveal general organization of camel (*C. dromedarius*) major *hsp70* cluster playing an important role in cellular and possibly whole body adaptation to extreme conditions. Such *hsp70* clusters linked with major histocompatibility complex (MHC) class III region have already been described in detail in several mammalian species including mice, rats, humans etc. [14]–[16]. Gene duplications leading to the three-gene cluster linked with MHC occurred early in the evolution because such a structure was described in frogs [17]. It was of significant interest to compare the organization of these clusters isolated from different organisms.

It is known that, while in humans there are 17 members of *HSP70* family genes located at different genomic sites, among all these genes only three *HSP70* family members form a cluster [14]. These three genes located next to the MHC region attracted much attention, probably because they are major players providing cellular response to high temperature and other extreme conditions [3], [8], [9]. In all mammalian species studied, two inducible members of this cluster *hsp70A1A* and *hsp70A1B* are found in tandem orientation separated by 7–9 kb [14]–[16]. The third gene termed *hsp70-like* (*hspA1L*) contains an intron shared by all mammalian species investigated and found in close vicinity to *hspA1A* gene located in inverse orientation. Inducible members of the cluster in all mammals studied so far contain heat shock elements (HSEs) and a canonical TATA box in their regulatory regions, while *hsp70A1L* gene does not contain either HSEs or canonical TATA in the promoter and, hence, other regulatory elements are apparently responsible for its constitutive expression in various tissues with a high level in testis.

Recently, the transcriptome of *C. dromedarius* has been annotated, which includes 23602 putative gene sequences searched for hits in the NIH Mammalian Gene Collection Project (http://mgc.nci.nih.gov) to identify matches to full-length cDNA sequences of *Homo sapiens*, *Mus musculus*, *Rattus norvegicus*, and *Bos taurus*. There are transcripts homologous to *hspA1A* and *hspA1B* in the transcriptome [18]. However, the genome of *C. dromedarius* has not yet been sequenced, and the general organization of the cluster and the promoter structure of *hsp70* family genes of this exceptionally thermoresistant organism are unknown.

Herein we provide the detailed structure of the *C. dromedarius hsp70* genes cluster, and compare regulatory regions of *hsp70* genes of this animal with available data on the organization of corresponding *hsp70* genes in other mammals studied in this respect. To this end, we obtained a lambda phage genomic library from *C. dromedarius*. Analysis of clones containing *hsp70*-homologous sequences enabled us to isolate and sequence the whole cluster containing the three genes belonging to the *hsp70* family. The analysis showed that the organization of *hsp70* cluster in camel is similar to that described in other mammals. Since we failed to detect specific features in regulatory regions of camel *hsp70* genes it is questionable whether expression of these genes may be implicated in extraordinary high heat tolerance of camel at the cellular and organism level.

## Results

### General organization of *C. dromedarius hspA1* cluster

**Figure 1:**
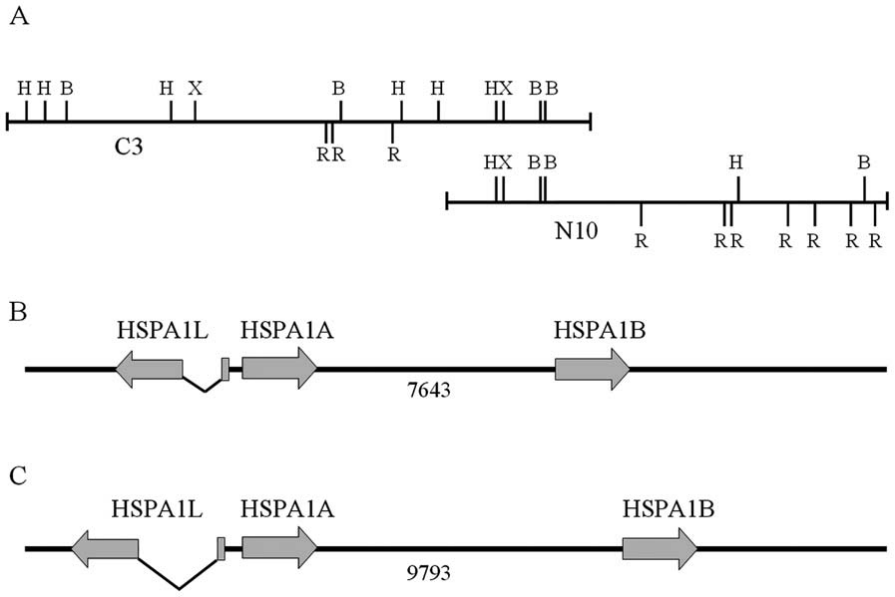
General organization of the *hsp70* cluster in camel and human. A – restriction maps of two overlapping recombinant phages, C3 and N10, used in the analysis (H – *Hind*III, X – *Xho*I, R – *Eco*RI, B – *Bam*HI). B – general structure of the *C. dromedarius HspA1* cluster. C – general structure of *H. sapiens HSPA1* cluster provided for comparison. The length of the intergenic region between *hspA1A* and *hspA1B* genes is given in bps.

In the course of this analysis, we have isolated 24 lambda clones after screening of a genomic library, and following preliminary PCR and restriction studies we have chosen two phages, which apparently include two overlapping halves of the investigated gene cluster (Figure 1A). The first phage, named “C3”, contains two genes, identified as orthologues of human *HSPA1L* and *HSPA1A*, located in inverted orientation, and a short 5′-fragment of the third gene homologous to human *HSPA1B*, separated by approximately 7.6 kb from the 3′-end of *hspA1A*. The second phage (“N10”) contains only *hspA1B*, with long 5′- and 3′-flanking regions, and overlaps with the phage C3 by 5′-flanking and 5′-coding regions of the *hspA1B* gene. The detailed organization of *hsp70* gene cluster of *C. dromedarius* was determined by subcloning and sequencing of these overlapping recombinant lambda clones isolated from genomic library, submitted into GenBank (Accession Number JF837187.1) and is depicted in Figure 1B. The arrangement of the cluster is in general similar to that described in human and other mammalian species. However, the distances between individual *hsp70* copies in camel are smaller and, hence, the whole cluster is more compact than in humans (Figure 1B, C).

### Southern analysis of genomic DNA

**Figure 2:**
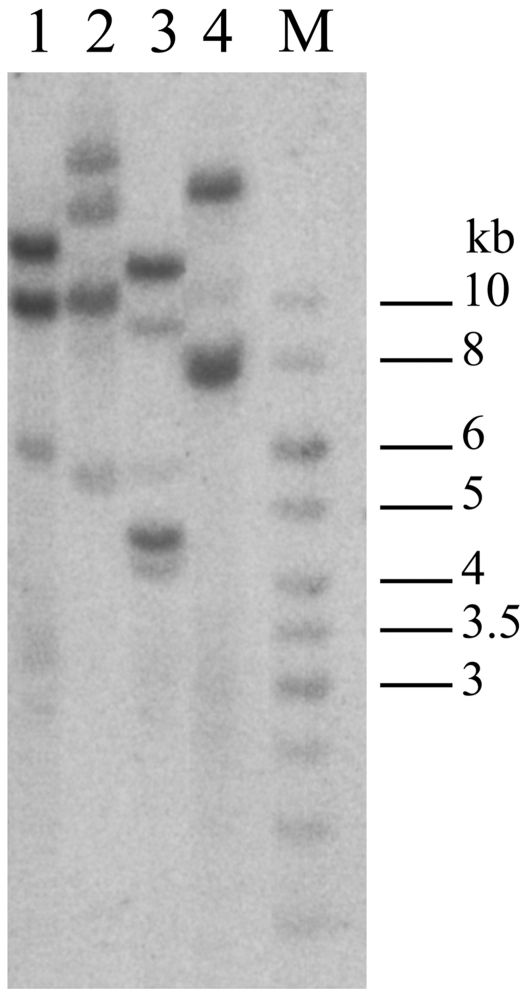
Southern blot of camel genomic DNA with PCR-probe to *hspA1A/B* genes. 1 – *Bam*HI, 2 – *Xba*I, 3 – *Xba*I/*Bam*HI, 4 – *Eco*RI. M – fragment length markers.

We have performed Southern blot analysis of *C. dromedarius* genomic DNA with *hspA1A* radioactively labeled probe to obtain independent data on the structure of the *hsp70A1* cluster and total number of *hsp70*-related genes. Figure 2 shows that restriction fragment length corresponds to the size of the fragments mapped within phages C3 and N10 exploiting the same restriction endonucleases, and hence these data fully corroborate our results based on phage analysis. Additional weakly hybridizing bands probably correspond to other *hsp70*-related genes, such as *hspA6* or *grp78*, which are located in other regions of the camel genome.

### Structure of ORF and UTR regions of *hspA1* genes of *C. dromedarius*

Detailed functional organization of the camel *hspA1* cluster including the boundaries of transcribed regions has been determined by phage DNA sequencing combined with the results of 3′- and 5′-RACE analysis with outward orientated primers specific to *hspA1A/B* or *hspA1L*.

ORFs of all three *hsp70* genes investigated have the same length equal to 1926 bp, including the stop codon. In the case of inducible *hspA1A* and *hsp70A1B* tandemly arranged genes, TAG serves as a stop codon, while in the case of *hspA1L*, which encodes a constitutively expressed protein, the stop codon is represented by TAA. *HspA1A/B* genes in camel do not have introns, resembling in this respect the corresponding genes from human and other mammals.

On the other hand, the *hspA1L* gene has two exons 173 bp and 2254 bp in length respectively. As in other mammals, the ORF in this gene starts in the second exon. While the boundaries of the *hspA1L* intron are identical in camel and other mammals, the intron length varies between species, due to the presence of transposable element insertions, microsatellite repeat expansions *etc*. In the camel, the intron is significantly smaller than in humans, *i.e.* 1169 bp *vs.* 2898 bp (Figure 1C). As expected, intron sequence conservation between species is much lower (60–70% identity) than that of coding and 5′-regulatory sequences.

ORFs of *hspA1A* and *hspA1B* are almost identical and differ by only four substitutions, one of which is silent. All differences in amino acid (a.a.) content between the two proteins are restricted to the N-terminus of the protein, which contains the ATP-binding domain (positions of a.a. substitutions: 55, 70 and 145) and, hence, may have some functional significance. In general, our data corroborate the previously demonstrated exceptionally high conservatism of *hsp70* genes in mammals as well as in other eukaryotic organisms [3], [4]. Interestingly, camel *hspA1A* contains only three substitutions in comparison with the corresponding *Bos taurus* protein. Table 1 summarizes the data on homology of camel *hspA1* genes (ORF) compared with the corresponding ORFs of other organisms. Characteristically, *hspA1L* ORF exhibits only 82% identity with *hspA1A* and *hspA1B* ORFs, while at the protein level the identity reaches almost 90%. It is noteworthy that in this case most of the substitutions are found in the C-terminal domain, corroborating previous results that demonstrated comparatively high variability of this domain in the entire *hsp70* gene family. Furthermore, 5′-UTRs of *hspA1A* and *hspA1B* exhibit 82% identity. Interestingly, 174 bp upstream from the actual ORF start, an additional ATG codon is located. However, in camel the next triplet is represented by the stop codon TAG and, hence, this ATG codon is apparently not functional. It is noteworthy that similar silenced ATG codons exist within UTRs of *hspA1A* described in other mammals, *e.g. Bos taurus* and *Bubalus bubalis* (Access. Nos. NM_174550.1 and HM025989.2). However, in all these cases the surrounding context of these ATG codons is not optimal for initiation, and probably these codons are also silent (Table 2). On the other hand, in contrast to camel, in *Bos taurus* and *Bubalus bubalis* there are no stop codons following this ATG, and uninterrupted ORFs 369 bp in length do exist. As expected, the 3′-UTRs of *C. dromedarius hspA1A* and *hspA1B* genes are more variable than 5′-UTRs and exhibit only 50% homology. In both *hspA1A/B* genes, the 3′-UTR contains a canonical polyadenylation signal (AATAAA), while the 3′-UTR of *hspA1L* contains an AGTAAA signal found also in orthologous genes of rats and humans. 3′-RACE analysis using specific primers (Table 3) demonstrated that all three *C. dromedarius hspA1* type mRNAs are effectively polyadenylated.

**Table 1:** Identity of camel *hspA1* genes with orthologues from other organisms.

Gene symbol	Species	Identity level in %
*A1A* (1923 bps)	*Bubalus bubalis*	96
	*Bos indicus*	96
	*Homo sapiens*	95
	*Xenopus laevis*	73
*A1B* (1923 bps)	*Bubalus bubalis*	96
	*Bos indicus*	96
	*Homo sapiens*	95
*A1L* (1923 bps)	*Sus scrofa*	94
	*Equus caballus*	94
	*Bos taurus*	93
	*Homo sapiens*	91
	*Xenopus laevis*	73
ψ *grp78* or BiP (1216 bps, partial CDS)	*Equus caballus*	96
	*Bos taurus*	96

ψ *Grp78* taken from different mammalian species exhibits 100% identity at amino acid level.

**Table 2:** The structure of *Hsp70* translation start and surrounding sequences in various organisms.

Species	ATG context
Kozak cons.	gcc**g** cca/gc**c** atg**g**a/ **c** t
Camel *hspA1A*	ggc**a** ca gg**c**atg**gc**g
Camel *hspA1B*	ggc**a** ca gg**c**atg**gc**g
Bubalus *hspA1A*	ggc**a** cc gg**c**atg**gc**g
Bos *hspA1A*	ggc**a** cc gg**c**atg**gc**g
Homo *hspA1A*	gga**a** cc gg**c**atg**gc**c
Homo *hspA1B*	ggc**a** cc gg**c**atg**gc**c
Camel *hspA1A* si	agc**t** tc ac**g**atg**ta**g
Bos *hspA1A* si	agt**t** gc gt**t**atg**tt**g
Bubalus *hspA1A* si	agc**t** tc ac**g**atg**tt**g

Nucleotides disturbing optimal context for translation initiation are marked by bold shrift. The position of ATG is underlined. si – upstream (silenced) ATG. Kozak cons. – consensus sequence optimal for translation initiation [29].

**Table 3:** List of primers used in the experiments.

Name	Sequence	Position	Application
CamORF1	CATCGGCATCGACCTGGGCA	5′-*hspA1A/B* inward	RT-PCR for detection of
CamORF2	CACTGATGATGGGGTTACACAC	3′-*hspA1A/B* inward	transcription,of *hspA1A/B* coding
			region amplification fragment
			for Southern hybridization
RT-1A	GATCAACGACGGAGATAAGCCG	5′-*hspA1A/B* inward	RT-PCR for detection
RT-2A	GCGTAAGACTCCAGGGCGTTC	3′-*hspA1A/B* inward	of transcription
RT-1L	AAAGCAGGTCAGGGAGAGCGA	5′-end of the *hspA1L*	
		second exon inward	
RT-2L	GGAGGGATTCCAGTCAGGTCA	3′-end of the *hspA1L*	RT-PCR for detection
		second exon inward	of transcription
5-RACE-A1	CGTGTTCTGCGGGTTCAGCG	5′-*hspA1A/B*	5′-RACE-analysis of *hspA1A/B*
5-RACE-A2	TGGTGCGGTTGCCCTGGTCG	outward	transcripts
3-RACE-A1	CTGGAGTCTTACGCCTTCAACA	3′-*hspA1A/B*	3′-RACE-analysis of *hspA1A/B*
3-RACE-A2	CCGACAAGAAGAAAGTGCTGGA	outward	transcripts
5-RACE-L1	TCAGTACCATAGAAGAGATTTCCT	5′-end of the *hspA1L*	5′-RACE-analysis of *hspA1L*
5-RACE-L2	ACCTTGGGCTTGCCTCCTTCA	second exon outward	transcript
3-RACE-L1	ATGAAGAGTGCTGTGAGTGATGA	3′-end of the *hspA1L*	
3-RACE-L2	AAGGGCAAGATTAGTGAGTCTGA	second exon	3′-RACE-analysis of *hspA1L*
3-RACE-L3	GAGAAAGGAATTGGAGCAGGTG	outward	transcript

### The structure of intergenic regions in *C. dromedarius hspA1* cluster

In camel, transcription start sites and polyA sites of *hspA1* genes were localized by 5′- and 3′-RACE analysis and comparison of RACE fragments with known phage DNA sequences.

The region between *hspA1A* and *hspA1L* genes is organized in *C. dromedarius* as an inverted repeat that constitutes only 414 bp, while in the human *hsp70* cluster the distance between the corresponding transcription starts is equal to 505 bp.

Promoters of *hspA1A* and *hspA1B* genes in *C. dromedarius* contain canonical TATA-boxes, while the promoter of *hspA1L* apparently belongs to the TATA-less type of promoters. In this respect, camel does not differ from close species (*Bos taurus*) and humans. In mice and rats, promoters of *hspA1A* and *hsp70A1B* genes do not include the classic sequence (TATAAA) but contain an alternative motif TTAAAG [15].

**Figure 3:**
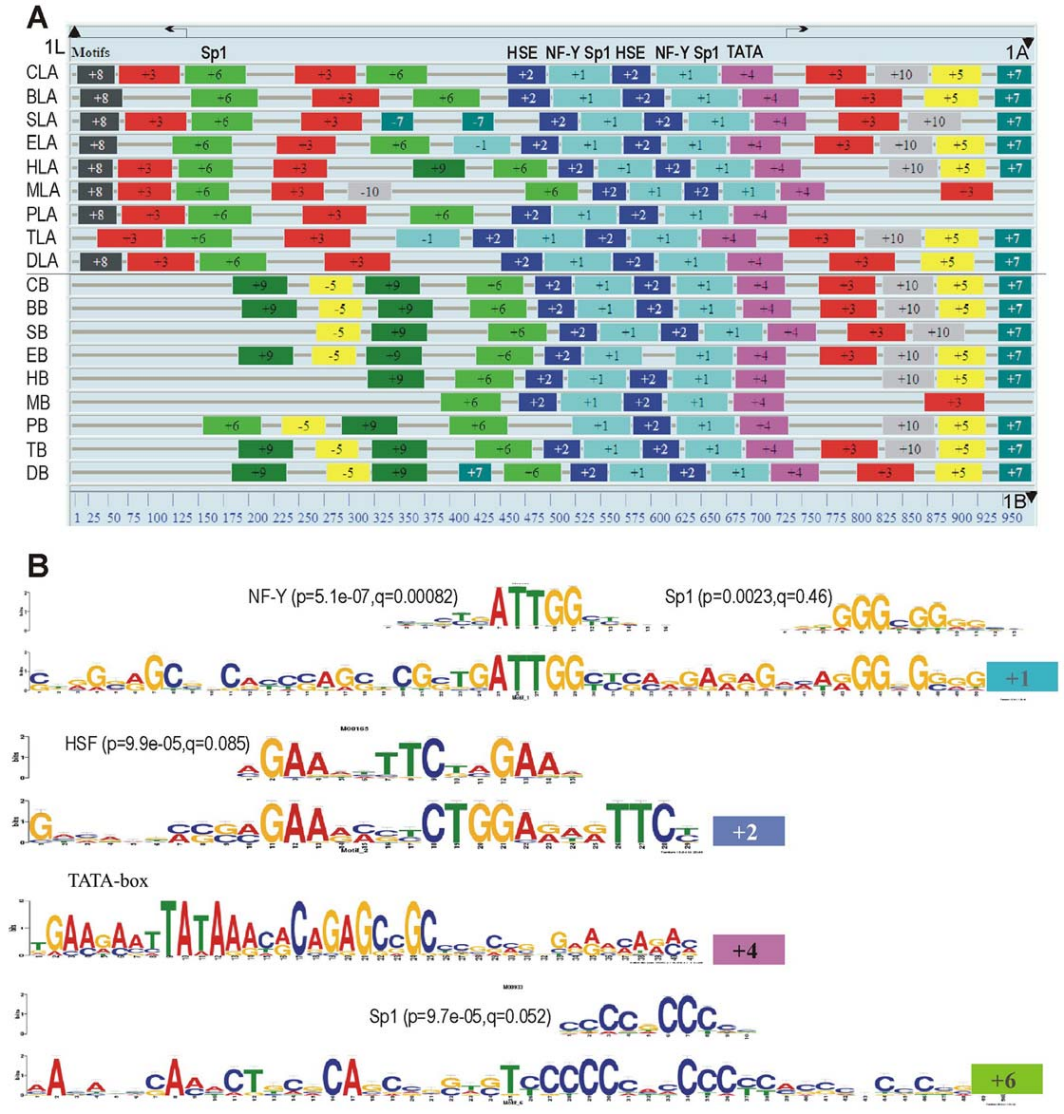
Comparison of 5′-regulatory regions of *hspA1* genes from camel and other mammals. (**A**) Identification of conserved motifs in the region between *hsp1L* and *hsp1A* (designated LA) and upstream of *hspA1B* (designated B) from *Camelus dromedarius* (C), *Bos taurus* (B), *Sus scrofa* (S), *Equus caballus* (E), *Homo sapiens* (H), *Mus musculus* (M), *Pteropus vampyrus* (P), *Tursiops truncatus* (T), and *Canis familiaris* (D). Transcription start sites are indicated by arrows, and ATG codons – by triangles. Intron sequences of *hsp1L* genes (located 16 bp upstream from the ATG codon, as indicated by a vertical dotted line) were removed to reduce sequence heterogeneity. Motifs are numbered in the order of identification by MEME, and numbers at the bottom indicate approximate base pairs in the alignment. (**B**) Matches between selected motifs from panel A and binding sites of known transcription factors in the TRANSFAC database identified by TOMTOM. Shown are the logos with the corresponding p- and q-values for each TF. The remaining motifs do not yield any matches to binding sites of known TFs.

We performed comparative analysis of promoter sequences of camel *hspA1* family group genes with the corresponding regulatory regions of orthologous genes from diverse mammalian species. Intergenic regions between the *hspA1A* and *hspA1L* inverted gene pair, as well as regions of comparable length upstream from *hspA1B*, were extracted from GenBank for *Bos taurus*, *Equus caballus*, *Sus scrofa*, *Homo sapiens*, *Mus musculus*, *Canis familiaris*, *Pteropus vampyrus*, and *Tursiops truncatus*, and aligned with the corresponding camel sequences by ClustalW. The 250-bp region immediately upstream of the TATA box of *hspA1A/B* genes exhibits the highest degree of conservation, likely reflecting the location of the most important regulatory elements. Outside of this region, the degree of sequence variability increases considerably in all species. Fig. 3 shows the results of conserved motif search done as described in Materials and Methods. Matches with highest significance are located in the 250-bp upstream region and correspond to HSE followed by NF-Y and Sp1 binding sites, an arrangement which is repeated twice in both *hspA1A* and *hspA1B* in each of the nine species shown in Fig. 3. For *hspA1L*, only the Sp1 binding site could be detected. The high degree of sequence conservation leaves little room for putative camel-specific regulatory elements in the 250-bp region upstream of the TATA box. Indeed, there are only five single-nucleotide differences shared between *hspA1A* and *hspA1*B from camel but not other mammals, however none of these apparently affect recognition of binding sites for known transcription factors, as may be seen from Fig. 3.

It is necessary to mention that promoter of camel *hspA1B* gene besides a couple of canonical HSEs contain three additional candidate HSEs in the interval from 900 to 2900 bps upstream of the TATA signal (Figure 3). One of these distant HSEs located at 2015 bps position from the transcription start represents a canonical structure GAAAGTTCCTGAA while the two other HSEs located at 1164 and 710 bps from the transcription start also contain three units with two substitutions in one of the units. These candidate HSEs may be responsible for differential expression of *hspA1A* and *hspA1B* genes in various tissues and under different temperature conditions.

The 3′-UTR regions of *hspA1A/B* also exhibit a high degree of conservation between species: for instance, there are only five nucleotide substitutions in the 3′-UTR of *hspA1A* from camel and from its closest relative *Lama pacos* (the 5′-regulatory region from this species is missing from the database). It remains to be seen whether any of these mismatches could influence the levels of *hspA1* expression in camel.

### Differential expression of *hspA1A*, *hspA1B* and *hspA1L* in camel cells of different origin

**Figure 4:**
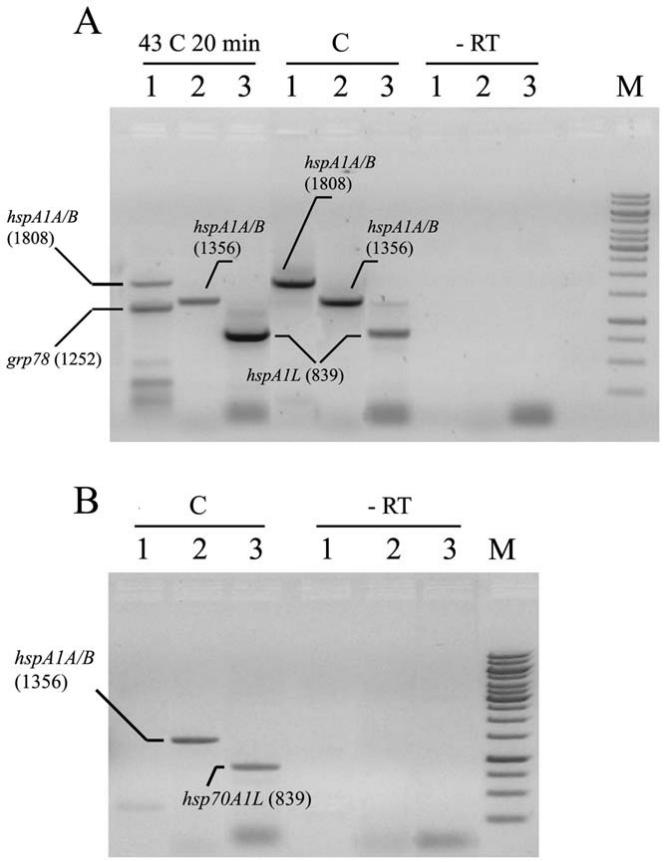
A – RT-PCR with total RNA from camel's blood. B – RT-PCR with total RNA from camel's heart muscle. 1 – primers CamORF1/2 to *hspA1A/B* genes and *grp78*, 2 – primers PT-1A and RT-2A to *hspA1A/B* genes, and 3 – primers RT-1L and RT-2L to *hspA1L* (see Table 3). RT – negative control of RT-PCR, samples without reverse transcriptase.

RT-PCR experiments exploiting primers homologous to *hspA1A*, *hspA1B* and *hspA1L* genes revealed the corresponding transcripts both in lymphocytes and heart muscle (Figure 4). Interestingly, transcription of all three members of the *hsp70* family, including the constitutively expressed *hspA1L*, has been demonstrated by RT-PCR experiments under normal physiological conditions (Figure 4). After temperature elevation (43°C, 20 min), an additional fragment 1253 bp in length has been detected with the first primer pair (Table 3). Subsequent sequencing demonstrated that this fragment corresponds to cDNA of *grp78* gene (JF837188.1), another glucose-regulated member of *hsp70* family (Figure 4).

As expected, transcripts of *hspA1A*, *hspA1B* and *hspA1L* are evident after HS using all three pairs of primers (Figure 4). The data accumulated in the course of RT-PCR studies have been corroborated by 5′- and 3′-RACE experiments. The latter approach revealed 5′- and 3′-untranslated fragments homologous to all three *hspA1*-type genes both in control (non-heated sample) and after temperature elevation, strongly suggesting that these genes are actively transcribed after heat shock and under normal physiological conditions. Since sequencing revealed characteristic differences in 5′- and 3′-UTRs of *hspA1A* and *hspA1B* genes, the presence of fragments homologous to both *hspA1A* and *hspA1B* enables us to conclude that both genes are expressed in lymphocytes.

An independent series of RT-PCR experiments with RNA isolated from heart muscle also detected significant signals with primers homologous to *hspA1A*, *hspA1B* and *hspA1L* genes (Figure 4B). Therefore, one may conclude that *hspA1A/B* and *hspA1L* genes are expressed to some extent both in camel lymphocytes and heart muscle tissue under normal conditions.

**Figure 5:**
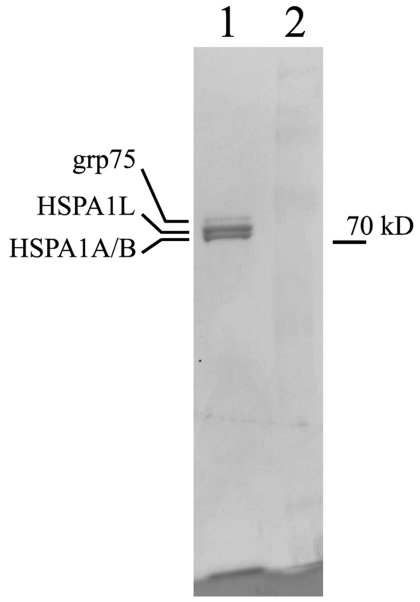
Hsp70 family proteins isolated from camel heart. The identity of proteins was determined by trypsin fingerprinting and microsequencing. Lane 2 – molecular weight marker.

This conclusion was subsequently confirmed and extended by analysis of proteins isolated from camel heart muscle (Figure 5) and identified by peptide sequencing (MALDI-fingerprinting). Molecular weights of hspA1A/B and hspA1L proteins, determined by electrophoresis and mass-spectrometry, precisely coincide with values obtained by conceptual translation (70.14 kD for hspA1A, 70.11 for hspA1B and 70.31 for hspA1L). The analysis enabled us to detect hspA1A/B proteins which are almost identical and, hence, can not be further resolved, as well as hspA1L and grp75 which also belongs to Hsp70 family and is expressed in mitochondria (data not shown). It is evident that hspA1A/B and hspA1L are represented by bands of similar intensity and, hence, corresponding loci are expressed approximately to the same extent in camel muscle under normal physiological conditions.

## Discussion

The *hsp70* gene family represents one of the most ancient and highly conserved protective systems present in all living organism studied so far. However, although individual members belonging to this family exhibit exceptionally high levels of homology even when distant organisms are compared, various phylogenetic groups of organisms exhibit strikingly different trends in the evolution and organization of *hsp70* gene clusters. Thus, in our previous studies on Diptera species including representatives of *virilis* group species of *Drosophila* and various species belonging to Stratiomyidae family, we have shown that the *hsp70* gene cluster is involved in active rearrangement processes, and closely related species and even geographical strains may differ by number and relative position of individual *hsp70* copies comprising the cluster [10], [19]. On the other hand, lizard species belonging to different families judging by Southern analysis preserve practically identical structural organization of the *hsp70* cluster [20].

In mammals, the major *hsp70* cluster has a very peculiar structure. In all mammalian species studied in this respect, the cluster contains two inducible members of *hsp70* family arranged as tandem pair, and one *hsp70*-like gene which is constitutively expressed with a high level in testis, and is located in inverse orientation (Figure 1). Interestingly, in various mammalian species, as well as in Xenopus, the *hsp70* cluster is linked with MHCIII locus [14], [17]. Linkage of these two vital loci is probably not random, taking into account similar structure of peptide binding sites of *hsp70* and MHC. Based on these results, it was suggested that MHC locus may have been formed by recombination between an immunoglobulin-like C-domain and the peptide-binding domain of *hsp70*-like genes at the early stages of vertebrate evolution [16], [17].

Along these lines, multiple recent studies implicate the Hsp70 family of proteins in modulation of the innate immune response of an organism [21]. High conservation of *hspA1* loci organization observed in the genomes of all mammalian species studied may provide specific chromatin conformation necessary for optimal functioning of vital MHC and *hspA1* loci involved in antigen processing and antigen presentation.

Furthermore, recently we have compared the ability of Hsp70 preparations of different origin to protect model animals from endotoxic shock and modify response of myeloid cells to lipopolysaccharide (LPS) challenge. Our experiments demonstrated that in several cellular models exogenous Hsp70 preparations isolated from camel's muscle were significantly more efficient than human recombinant Hsp70 in innate immunity modulation and stimulation of endogenous protective mechanisms [22].

Our analysis of the *hspA1* cluster in the camel did not reveal camel-specific features, either in general organization of the cluster or in the structure of regulatory regions of *hsp70* genes. The high degree of sequence conservation leaves little room for putative camel-specific regulatory elements in the promoters studied. It is noteworthy, however, that we detected three additional non-canonical but possibly functional HSEs in the regulatory region of *hsp70A1B*, which may account for higher levels of *hsp70* expression observed for camel cells in comparison with comparable cells of other organisms [6], [13]. Furthermore, a few substitutions observed in camel's hspA1-group proteins may lead to higher stability of the proteins.

Although it is widely accepted that Hsp70 plays an important role in thermoresistance, we can not exclude other factors that may contribute to heat tolerance exhibited by camel cells. Thus it was demonstrated that expression levels of Akt, an important prosurvival kinase, are uniform in camel fibroblasts, irrespective of the temperature, while stress activated kinase (Jnk) was induced in these cells by temperature elevation [13]. It is also possible that activated heat shock transcription factor (HSF1) exists in camel cells at normal physiological temperatures, providing constitutive expression of heat inducible members of Hsp70 family, as was previously described for desert lizard species [20].

In accordance with this supposition, our RT-PCR and 5′- and 3′-RACE experiments clearly demonstrated that all *hspA1*-group genes are expressed both after heat shock and under normal physiological conditions. These results corroborate our previous results showing that temperature elevation increases the level of constitutively expressed Hsp70 in camel cells [23].

It is not clear, however, what regulatory motifs are responsible for high level of expression of camel constitutive *hsp70* genes lacking HSEs after HS.

General structure of *hsp70* cluster of camel is very similar to the organization of *hsp70* clusters described in other mammalian species described so far. A high degree of evolutionary conservation observed for the *hsp70* cluster always linked with MHC locus in mammalian species suggests an important role of such organization for coordinated functioning of these vital genes. All three *hsp70* genes comprising the cluster are actively transcribed in different camel tissues not only after heat shock, but under normal physiological conditions as well, and may account for tolerance of camel cells to extreme environmental conditions.

Our data strongly suggest that the three *hspA1* genes are likely to functionally interact with each other and probably with linked MHC locus in many processes, both positively and negatively, including tolerance to various deleterious factors and innate immunity modulation. Their role in these and other processes should be uncovered in future by exploring various cellular and animal models, enabling to directly investigate the interactions between these vitally important genomic loci.

## Materials and Methods

### Animals

All procedures involving live animals were reviewed and approved by the Animal Care and Use Committee of The Severtsev Institute of Problems of Evolution and Ecology RAS where animals were housed. All animal experiments were performed in accordance with the guidance of the National Institutes of Health for care and use of laboratory animals, NIH Publications No. 8023, revised 1978. Certification for this project has been provided by Animal Care and Use Committee of Severtsev Institute of Problems of Ecology and Evolution RAS. (Protocol No. 229/131).

### DNA isolation, genomic library construction, screening and clone analysis

Genomic DNA was isolated from *C. dromedarius* frozen heart muscle by standard method with phenol/chloroform extraction described in [24]. Frozen heart muscle was obtained as a by-product by our expedition to Ashhabad (Turkmenistan) in 2005 from a meat factory where camel meat is produced for food industry.

Genomic library was prepared by partial Sau3A digestion of camel DNA with subsequent ligation into the *Bam*HI site of lambda Dash phage arms (Stratagene). Before ligation, restriction mixture was separated by ultracentrifugation in sucrose gradient for removal of short restriction fragments, so that the resulting fraction contained fragments 14–23 kb in length that were used for cloning. Gradient parameters were: 10–40% sucrose in 1 M NaCl, 20 mM Tris-HCl pH 8.0 and 5 mM EDTA with ultracentrifugation at 26,000 g for 24 hours. Ligated DNA was packaged into phage particles using lambda packaging extracts Gold (Stratagene). Recombinant phages were selected, amplified and screened using *E. coli* XL-Blue MRA (P2) host strains. For screening of the genomic library, a fragment of human *HSPA1A* cDNA obtained by PCR amplification with specific primers indicated in Table 3 was used as a probe after random prime labeling. Positive recombinant phages containing the presumptive camel *hsp70* genes were investigated by restriction analysis and hybridization, and fragments of interest were subcloned into the phagemid pBluescript SK+ for sequencing. Clones were sequenced using Sequenase II (Amersham) and ABI 377 sequencer. Sequences were assembled manually and aligned using NCBI Blast and Vector NTI.

### Southern blotting

Southern blot analysis of *C. dromedarius* genomic DNA was performed as described [24]. Briefly, ten micrograms of each DNA sample was digested with different restriction endonucleases. After agarose gel electrophoresis, gels were treated for 15 min in 0.25 M HCl and then incubated twice in denaturing buffer (1.5 M NaCl, 0.5 M NaOH) for 30 min. After 30 min incubation in neutralization buffer (1.5 M NaCl, 0.5 M Tris-HCl pH 7.5), gels were capillary-blotted onto nylon membranes and fixed by UV cross-linking using the UV Stratalinker 2400 (Stratagene). Hybridization and washing temperature was 65°C. To detect *hsp70*-containing sequences, the Southern blot was probed with the PCR-fragment of previously cloned *C. dromedarius hspA1A* gene obtained with primers indicated in Table 3.

### Isolation of lymphocytes from venous blood and heat shock conditions

The camel blood was obtained from the jugular vein of adult animal and EDTA was immediately added to prevent coagulation. The blood cells were separated on Ficoll gradient as described [25]. Lymphocyte fraction was isolated, washed in 3X PBS by spinning at 100 rpm for 10 min to get rid of thrombocytes, and heat shocked in a Petri dish (43°C 20 min) when necessary.

### RNA isolation and RT-PCR and RACE analysis

Total RNA from heat shocked and control lymphocytes and heart muscle was prepared by the standard method with TRIZOL (Invitrogen). Synthesis of first strand of cDNA from total RNA and subsequent amplification of specific interval cDNA fragments were performed using MINT cDNA kit (Evrogen) in accordance with manufacturer's instructions. For specific 5′- and 3′-end amplification (RACE analysis), outward primers to 5′- and 3′-fragments of *hspA1A/B* and *hspA1L* coding regions were used (Table 3). PCR conditions depended on primer annealing time and temperature. All reactions contained 1.25 units of Encyclo DNA polymerase (Evrogen) per probe, 1.5–2.5 mM MgCl_2_, 0.2 mM of each dNTP, suitable quantity of DNA and 10 pM of each primer in 50 µl (total volume). Primers specific for different camel *hsp70* genes are given in Table 3. The resulting PCR fragments were cloned into pTOPO-II vector (Invitrogen) and sequenced using plasmid-specific primers. In all RT-PCR experiments, probes containing all components but lacking reverse transcriptase were routinely used as negative controls.

### Purification of camel proteins belonging to Hsp70 family and fingerprinting analysis

200 grams of heart muscle tissue were homogenized in low salt buffer (20 mM NaCl, 20mM Tris pH 7.5, 0.1 mM EDTA, 0.1% Triton X-100) and centrifuged at 4000 g 45 min. Supernatant was filtered through filter paper and placed onto chromatography column with DEAE sepharose (GE), and Hsp70 family proteins were partially purified as described [26] with slight modifications. Three isolated proteins belonging to Hsp70 family were separated by standard SDS-PAGE method and identified by trypsin fingerprinting and a database search as previously described [27]. Protein identification was done by trypsin fingerprinting using surface-enhanced laser desorption ionization-time-of-flight-mass spectrometry (MALDI-TOF-MS) followed by NCBI database search using Profound search engine. Proteins used in these experiments were obtained as gel slices after SDS-PAGE electrophoresis stained with Coomassie Blue (G-250). In-gel trypsin proteolysis was performed as described in [7].

### Sequence analysis

Sequences from genome databases included into the analysis were as follows:

*Bos taurus* (cow) heat-shock 70-kilodalton protein 1A (*hspA1A*) gene, *hspA1A-D* allele, complete cds GenBank: AY149619.1

*Bos taurus* heat-shock 70-kilodalton protein 1A (*hspA1A*) and heat-shock 70-kilodalton protein 1B (*hspA1B*) genes, complete cds GenBank: AY149618.1AY149618.1

*Sus scrofa* (pig) DNA sequence from clone PigI-711D2, complete sequence GenBank: AL773559.16

*Equus caballus* (horse) *hspA1A*-*A1L* GenBank WGS AAWR02009906.1:9200-12000, contig 2.9905, and *hspA1B* AAWR02009906.1:20000-22000

*Homo sapiens* (human) heat shock 70 kDa protein 1-like (*HSPA1L*), RefSeqGene on chromosome 6 NCBI Reference Sequence: NG_011855.1

*Mus musculus* (mouse) DNA sequence from clone RP24-186I6 on chromosome 17, complete sequence GenBank: CU457784.5

*Pteropus vampyrus* (large flying fox) *hspA1A-A1L* GenBank WGS ABRP01095227, contig 1.95226, and *hspA1B* ABRP01287833, contig 1.287832

*Tursiops truncatus* (bottlenosed dolphin) *hspA1A-A1L* GenBank WGS ABRN01328970 contig 1.328969, and *hspA1B* ABRN01328973, contig 1.328972

*Canis familiaris* (dog) *hspA1A-A1L* GenBank WGS NW_876254:1286040-1288108 chromosome 12 contig, and *hspA1B* NW_876254:1300000-1302000.

Regulatory regions of *hsp70* genes were searched for common motifs by MEME, and identification of matches to known transcription factors has been performed in the TRANSFAC and JASPAR databases by TOMTOM in the MEME suite (http://meme.nbcr.net) [28].
